# Localization of Buried Polyethylene Gas Pipelines Using Compressional Wave Migration Stacking Imaging

**DOI:** 10.3390/s25185826

**Published:** 2025-09-18

**Authors:** Ao Zhang, Junfeng Liao, Disheng Zhong, He Li, Ping Han, Zhenting Song

**Affiliations:** 1 School of Mechanical Engineering, Shenyang Jianzhu University, Shenyang 110168, China; zhangao2902@sjzu.edu.cn (A.Z.);; 2 Key Laboratory of Vibration and Control of Aero-Propulsion System, Ministry of Education, Northeastern University, Shenyang 110819, China

**Keywords:** PE pipeline localization, compressional wave, stacking imaging, pipeline excitation method, wavelet decomposition

## Abstract

Buried polyethylene (PE) gas pipelines are widely used in urban construction. Precise localization of these pipelines is essential for regular maintenance. To address the issue of insufficient accuracy in existing localization techniques, this paper proposes a localization method based on compressional wave migration stacking imaging. The pipeline excitation approach is utilized to avoid interference from reflected waves, and the wavelet decomposition method is employed to suppress environmental noise and improve the signal-to-noise ratio. A pipe–soil coupling model was established using COMSOL6.3 Multiphysics to analyze elastic wave propagation induced by pipeline excitation. The results revealed a distinct velocity disparity between compressional wave and shear wave, with compressional wave velocity exhibiting significant superiority. Leveraging this propagation characteristic, we propose a novel pipeline localization method based on compressional wave migration stacking imaging. The method’s accuracy was validated through simulations and field experiments. Experimental results showed that the horizontal localization error was below 0.5%, and the depth error was below 4.25%, demonstrating a reliable localization accuracy. Furthermore, the pipeline direction was intuitively identified using 3D imaging technology, effectively distinguishing it from other foreign objects in the soil. This study provides a high-precision, low-interference solution for the trenchless detection of buried PE pipelines in complex soil environments.

## 1. Introduction

Pipelines play a vital role in transporting, collecting, and distributing water, oil, gas, and other liquid resources [1]. As a crucial component of urban infrastructure, buried pipelines form interconnected underground networks, enhancing urban space utilization efficiency and holding significant importance for urban development [2,3]. Polyethylene (PE) pipelines are extensively used in urban environments due to their excellent impact and corrosion resistance [4]. However, PE pipelines buried long-term in complex soil environments can suffer damage or fracture due to ground settlement or uneven installation, which can lead to leakage of their contents and potentially cause explosions and fires [5,6,7]. Additionally, incomplete pipeline construction information and immature PE pipeline localization techniques pose challenges for localization [8]. Therefore, to facilitate regular maintenance of PE pipelines, locating them rapidly and accurately is an urgent problem to address.

Traditional pipeline localization methods primarily rely on metal detectors [9], tracer wires [10], and ground-penetrating radar (GPR) [11]. Due to the non-conductive and non-magnetic properties of PE pipes, traditional metal pipeline localization methods cannot be applied [12]. The tracer wire method utilizes the principle of electromagnetic induction, in which a metal tracer wire is laid along the pipeline surface, and the pipeline route is determined by detecting the electromagnetic induction current emitted by the wire [13]. However, tracer wires are prone to breakage and corrosion in complex soil environments, which can lead to signal loss, and their electromagnetic induction signals are susceptible to interference, resulting in inaccurate localization results. As a non-destructive testing technique, GPR involves transmitting electromagnetic waves underground and analyzing the reflected echoes received at the surface, and it is commonly used to map subsurface profiles and features [14,15]. However, the target echoes received by GPR are often affected by clutter, and soil moisture content significantly influences the propagation, attenuation, and reflection of electromagnetic waves, resulting in substantial deviations in localization results [16,17]. In recent years, the rapid development of acoustic localization technology has provided new solutions to the challenge of locating buried PE pipelines. Acoustic signals are immune to interference from environmental electromagnetic waves during propagation, making this approach a reliable method for buried PE pipeline localization [18].

Some scholars have conducted in-depth research on acoustic detection methods for underground PE pipelines. The main methods include point vibration measurement [19], pipeline excitation method [20], and elastic wave imaging [21]. Muggleton J M et al. [22] used low-frequency vibro-acoustics via point vibration measurement to locate buried PE pipelines. The results showed that the resonance characteristics of ground structures can effectively reflect the presence of buried objects, and preliminary experimental results also verified the feasibility of this method. Although this technology has advantages in measuring and analyzing speed, it is currently immature, and its detection depth is limited, making it unable to accurately locate PE pipelines. Yan B Y et al. [23] injected specific frequency pulse acoustic signals into buried PE pipelines in their positioning experiments, and analyzed the amplitude of the specific frequency signals propagating to the ground through fast Fourier transform. Due to the rapid attenuation of the pulse signal, the horizontal position of the pipeline was found through the maximum amplitude. Qi Y S et al. [24] demonstrated through the principle of acoustic waveguide and finite element analysis that resonance occurs when sound waves propagate in pipelines. The author selected the signal amplitudes at the two resonance frequencies with the minimum attenuation and fitted them to construct a frequency selective pipeline depth localization algorithm, effectively estimating the burial depth of the pipeline. Zhang A et al. [25] proposed a pipeline excitation method based on acoustic attenuation for locating buried PE pipelines. Using the low-order resonance frequency of the pipeline as the excitation frequency, they derived a mathematical model for acoustic wave amplitude decay with propagation distance. Through the proposed method, the burial depth of the PE pipeline could be estimated, and the results were validated effectiveness through field experiments. Zhang H et al. [26] proposed a pipeline excitation method combining cross-correlation time-delay localization technology with an elliptic equation, which accurately calculated the depth of buried PE pipelines and was verified through FEA simulation and field experiments. The pipeline excitation method requires the injection of acoustic waves into the buried pipeline via a pressure regulating box. Since acoustic waves propagate slowly and attenuate significantly in non-metallic materials, this method is susceptible to environmental interference. Elastic wave imaging can be subdivided into three types: compressional wave imaging, shear wave imaging, and surface wave imaging. Papandreou B et al. [27] used reflected compressional waves to detect shallow-buried underground objects. The authors calculated the envelope of the cross-correlation function between the ground-measured signal and the excitation signal, and summed the envelopes to generate cross-sectional images. Muggleton J M et al. [28] excited shear waves on the ground surface using low-frequency acoustic vibration technology, and calculated the generalized cross-correlation function coefficients between the shear waves and the excitation signal. Through stacking imaging, the pipeline position in a noisy experimental environment could be detected effectively. Scott W R et al. [29] developed a system using seismic surface waves and non-contact displacement sensors, to detect shallowly buried landmines. Their research showed that this method can effectively locate underground objects but is only suitable for detecting shallowly buried objects. Qi Y et al. [30] proposed a localization method for buried PE pipelines using three-dimensional time-domain stacking of reflected compressional waves. The authors positioned the excitation source directly above the pipeline to reduce shear wave interference. Later, Qi Y et al. [31] proposed a back projection algorithm (BPA) that imaged the target area by coherently accumulating response values from multiple measurement points, improving the resolution and localization accuracy of compressional wave imaging. Cui X et al. [32] generated two-dimensional and three-dimensional acoustic field maps of underground pipelines by stacking cross-correlation coefficients of signals between sound sources and detector arrays. The authors adopted a strategy of “multiple points transmitting, multiple points receiving, and cross-correlation coefficient stacking” to suppress clutter interference in the imaging results. According to the above studies, these elastic wave imaging methods require filtering out surface waves and direct waves before imaging. Furthermore, these methods are not suitable for complex scenarios with multiple pipelines laid in close proximity.

Although acoustic localization technology can be effectively applied to the detection of buried PE pipelines, it is still in its early stage, and the localization accuracy and effectiveness require improvement. To accurately locate buried PE pipelines and reduce the impact of environmental noise, further in-depth exploration is necessary. Elastic wave imaging can simultaneously display the depth and horizontal position of a pipeline clearly through soil cross-sectional imaging. However, the process of removing surface waves and direct waves is complex, and the energy of reflected waves is weak and susceptible to ground noise, which degrades imaging results. The pipeline excitation method can employ specific-frequency excitation signals to stimulate the pipeline, turning it into an underground sound source, thereby enhancing signal strength. This paper adopts a combined approach using both the compressional wave imaging method and the pipeline excitation method, enhancing acoustic signal strength while eliminating the need to remove surface waves and direct waves. In this paper, Section 2 elaborates on the fundamental equations of elastic waves, and derives the wave velocity equations for compressional and shear waves. Also, the cross-correlation and wavelet decomposition theory are introduced, providing theoretical support for subsequent experimental signal processing. In Section 3, the simulation analysis is conducted to verify the theoretical results. Meanwhile, the propagation mechanism of elastic waves in buried PE pipelines under vertical excitation is analyzed, and an algorithm for compressional wave migration stacking imaging is proposed. The proposed algorithm is then used for localization simulation analysis of an underground PE pipeline. Section 4 conducts field experiments on pre-buried pipelines. The collected signals undergo wavelet decomposition and reconstruction, and the reconstructed signals can clearly show compressional waves and shear waves. To improve imaging accuracy, the reconstructed signals are processed using the RMS (Root Mean Square) envelope extraction technique. The wave velocity in the soil is tested using the cross-correlation algorithm, and the processed compressional wave signals is used for migration stacking imaging, which accurately locates the position of the pre-buried pipeline. Both the simulation results and experimental results can locate the buried pipeline accurately, verifying the proposed positioning method. Also, by applying three-dimensional stretching to the two-dimensional image, the direction of the buried PE pipeline can be determined, further verifying the method’s feasibility.

## 2. Theory

### 2.1. Basic Theory of Elastic Waves

Generally, soil is a three-phase body, consisting of solid, liquid, and gas phases. Studying the propagation characteristics of elastic waves in such a medium is a highly complex process. In a PE pipeline–soil coupling model, the propagation characteristics of elastic waves are influenced by various factors such as the internal medium of the pipeline, pipeline dimensions, and wall thickness. This research primarily explores how to utilize the propagation characteristics of elastic waves to locate buried PE pipelines. It is assumed that both the soil and the PE pipeline are isotropic linear elastic materials. The governing equations for elastic wave propagation in a linear elastic medium are described by the following three Equations [30]:

(1) $$\rho \frac{\partial^{2}u}{\partial t^{2}}=\nabla \cdot \sigma$$

(2) $$\varepsilon =\frac{1}{2}(\nabla u+{\left(\nabla u\right)}^{T})$$

(3) $$\sigma =\lambda \left(\mathrm{tr}\varepsilon \right)I+2\mu \varepsilon$$

where *ρ* is the density of medium; **u** is the displacement vector; ∇ is the gradient operator; *σ* is the stress tensor; *t* is time; *ε* is the strain tensor; *λ* and *μ* are Lamé coefficients; tr*ε* is the trace of the strain tensor; **I** is the identity tensor. Among the above equations, Equation (1) is the equation of motion; Equation (2) describes the relationship between stress and strain, known as Hooke’s law; Equation (3) describes the relationship between strain and displacement.

Combining these three equations yields the elastic wave equation expressed in terms of displacement. For isotropic media, this can be further simplified as [33]:

(4) $$(\lambda +\mu )\nabla (\nabla \cdot u)+\mu \nabla^{2}u=\rho \frac{\partial^{2}u}{\partial t^{2}}$$

According to Helmholtz’s theorem, the displacement vector u can be expressed as the sum of the gradient of a scalar potential **Φ** and the curl of a vector potential h [33]:

(5) u=∇Φ+∇h

where **Φ** represents the volumetric strain; ∇**Φ** represents compressional wave (P-wave); **h** represents the rotation vector; ∇**h** represents shear wave (S-wave).

Taking the divergence of both sides of Equation (4) yields the wave equation for the compressional wave [30]:

(6) $$\left\{\begin{array}{l} \nabla^{2}\Phi =\frac{1}{c_{p}^{2}}\overset{\cdot \cdot }{\Phi }\\ c_{p}=\sqrt{\frac{\lambda +2\mu }{\rho }} \end{array}\right.$$

where *c_p_* is the propagation velocity of the compressional wave.

Similarly, taking the curl of both sides of Equation (4), yields the wave equation for the shear wave [30]:

(7) $$\left\{\begin{array}{l} \nabla^{2}h=\frac{1}{c_{s}^{2}}\overset{\cdot \cdot }{h}\\ c_{s}=\sqrt{\frac{\mu }{\rho }} \end{array}\right.$$

where *c_s_* is the propagation velocity of the shear wave.

For isotropic linear elastic media, the Lamé coefficients *λ* and *μ* can be expressed as [33]:

(8) $$\lambda =\frac{E\upsilon }{\left(1+\upsilon \right)\left(1-2\mu \right)},\mu =\frac{E}{2\left(1+\upsilon \right)}$$

where *E* is the Young’s modulus of the medium; *υ* is the Poisson’s ratio.

Substituting Equation (8) into Equations (6) and (7) gives:

(9) $$c_{p}=\sqrt{\frac{E}{\left(1-2\upsilon \right)\left(1+\upsilon \right)\rho }}$$

(10) $$c_{s}=\sqrt{\frac{E}{2\left(1+\upsilon \right)\rho }}$$

Since Poisson’s ratio typically ranges from 0 to 0.5, it follows that *c_p_* > *c_s_*.

### 2.2. Cross-Correlation Theory

For two time-domain signals $y_{1}(t)$ and $y_{2}(t)$, the cross-correlation function $R_{y_{1}y_{2}}(\tau )$ is defined as [34]:

(11) $$R_{y_{1}y_{2}}(\tau )={\int_{-\infty }^{\infty }y}_{1}(t)y_{2}(t+\tau )dt$$

where τ represents the time delay between signals $y_{1}(t)$ and $y_{2}(t)$. The cross-correlation function $R_{y_{1}y_{2}}(\tau )$ reflects the similarity of signal $y_{1}(t)$ shifted relative to $y_{2}(t)$. When $y_{1}(t)=y_{2}(t-\Delta t)$, the peak of $R_{y_{1}y_{2}}(\tau )$ occurs at τ=Δt, where Δt represents the time delay and it can be derived as:

(12) $$\Delta t=\arg\underset{\tau }{\max}R_{y_{1}y_{2}}(\tau )$$

If two sensors receive the same elastic wave signal, the difference in propagation paths causes a time delay Δ*t*. By determining Δ*t* using the cross-correlation function, the wave velocity c can be calculated [35]:

(13) $$c=\frac{d}{\Delta t}$$

where *d* represents the distance between two sensors.

### 2.3. Wavelet Decomposition Theory

Wavelet analysis characterizes local signal information in both the time and frequency domains, making it highly suitable for processing non-stationary and non-linear signals. Wavelet decomposition and reconstruction techniques, as extensions of wavelet analysis, decompose a signal into high-frequency and low-frequency components at different scales. These techniques filter out noise and extract useful signals. The continuous wavelet transform (CWT) of a signal x(t) is expressed as [36]:

(14) $$W_{x}(a,b)=\frac{1}{\sqrt{\left|a\right|}}\int_{-\infty }^{\infty }x(t)\psi^{*}(\frac{t-b}{a})dt$$

where ψ(⋅) is the wavelet function (mother wavelet); $\psi^{*}(\cdot )$ represents the complex conjugate of the mother wavelet function; *a* represents the scale parameter; *b* represents the translation parameter. The CWT achieves time–frequency localized analysis of signals through scaling and translation but has high computational complexity, making it suitable for theoretical research. The discrete wavelet transform (DWT) improves computational efficiency by discretizing the parameters, facilitating practical applications. The DWT consists of two steps: signal decomposition and reconstruction.

The signal x[n] is decomposed into low-frequency and high-frequency components:

(15) $$A_{j}[k]=\sum_{n}h[n]x[2k-n]$$

(16) $$D_{j}[k]=\sum_{n}g[n]x[2k-n]$$

where *A_j_* is the low-frequency coefficients; *D_j_* is the high-frequency coefficients; h[*n*] is the low-pass filter; g[*n*] is the high-pass filter.

The low-frequency and high-frequency coefficients are recombined to reconstruct the original signal X[*n*]:

(17) $$X[n]=\sum_{k}A_{j}[k]\phi_{j,k}[n]+\sum_{k}D_{j}[k]\psi_{j,k}[n]$$

where $\phi_{j,k}[n]$ is the scaling function and $\psi_{j,k}[n]$ is the wavelet function.

This study locates pipelines by introducing elastic waves onto a buried pipeline and utilizing their propagation characteristics in soil, providing a trenchless detection method for buried PE pipelines. Before detection, detailed theoretical analysis and simulation modeling of elastic wave propagation in soil are necessary to propose a suitable detection method.

## 3. Simulation Analysis

### 3.1. Simulation Analysis of Elastic Wave Propagation

**Figure 1:**
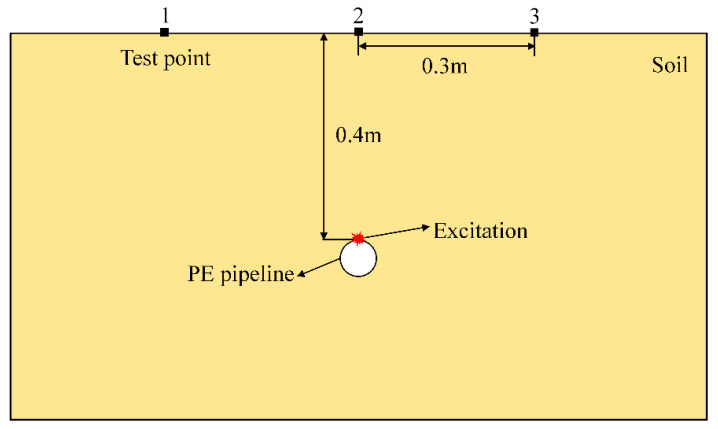
Two-dimensional simulation model of a buried PE pipeline.

**Figure 2:**
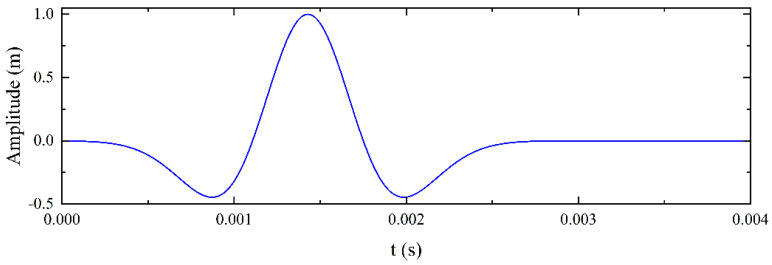
Waveform of the Ricker wavelet.

The COMSOL Multiphysics finite element simulation software was used to investigate the propagation characteristics of elastic waves in a pipe–soil coupling system. Although the viscoelasticity and damping of soil can affect the propagation and attenuation of waves, for the sake of simplifying research, it is assumed that the soil is a linear elastic material [31]. Due to the need to analyze the vertical component of elastic wave signals, a two-dimensional simulation model is established as shown in Figure 1. In the simulation, the commonly used ordinary sand is selected as the soil material, with a density of 1985 kg/m^3^, a Young’s modulus of 8.3 × 10^7^ Pa, and a Poisson’s ratio of 0.3 [37]. The pipeline material is high-density polyethylene (HDPE) with a density of 900 kg/m^3^, a Young’s modulus of 2 × 10^9^ Pa, and a Poisson’s ratio of 0.4. In order to reduce the influence of elastic wave reflection, the boundary conditions of the two-dimensional model (except for the surface of the soil) are set to low reflection boundaries. The grid size specifications are: maximum cell size of 0.015 m; minimum cell size of 0.00003 m; and maximum cell growth rate of 1.1. The curvature factor is 0.2. To enhance the elastic wave signal strength, the pipeline’s resonance frequency (700 Hz) was used as the excitation signal frequency based on the previous research [25]. The excitation signal was a 700 Hz pulse, modeled as a Ricker wavelet, which is known for its high sensitivity and penetration depth. Its waveform is shown in Figure 2.

**Figure 3:**
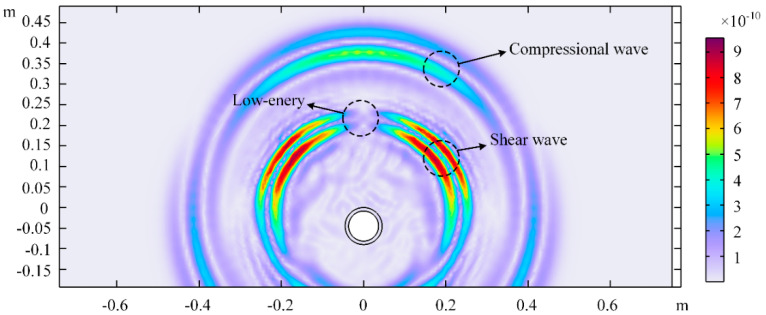
Displacement cloud map at t = 0.00215 s.

**Figure 4:**
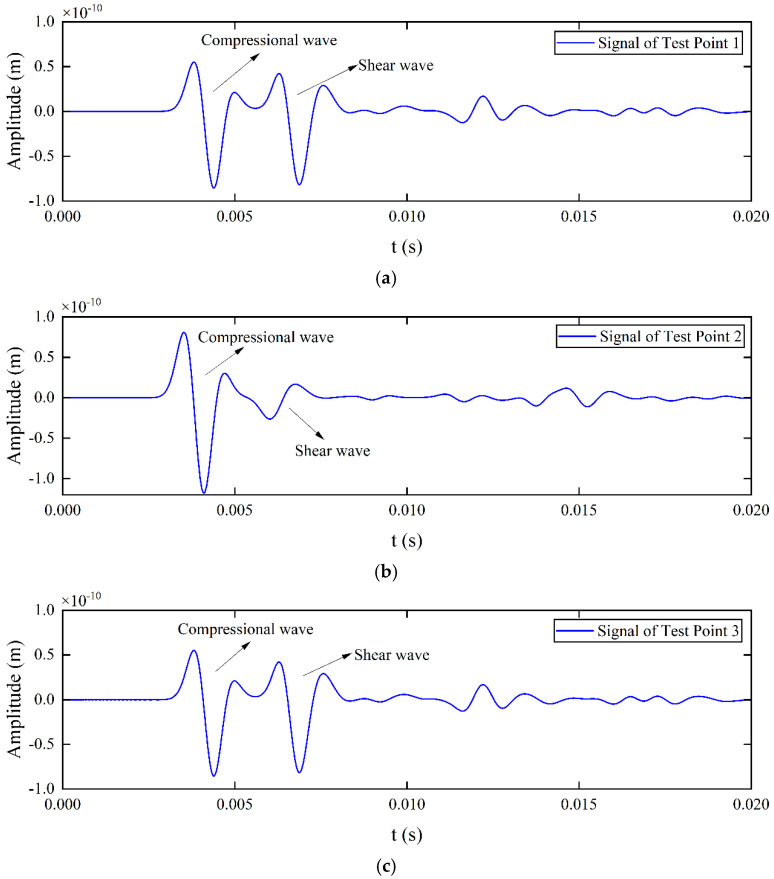
Signals measured at three test points in the simulation. (**a**) Signal of Test Point 1; (**b**) Signal of Test Point 2; (**c**) Signal of Test Point 3.

Displacement distribution (y-component) cloud maps in the soil domain at a specific time were plotted to observe elastic wave propagation, as shown in Figure 3. When the excitation source stimulates the pipeline surface, P-waves (compressional waves) and S-waves (shear waves) are generated. It can be seen that the P-wave velocity is significantly higher than the S-wave velocity. Due to signal attenuation characteristics, the S-wave amplitude is larger than the P-wave amplitude at this location. A low-energy zone for S-waves can also be observed directly above the excitation source, which is not suitable for detection when the sensor is arranged near this direction. The signals measured at the three test points in Figure 1 are shown in Figure 4. Since P-wave velocity exceeds S-wave velocity, the P-wave and S-wave in the signals can be clearly distinguished. The S-wave signal amplitude is very small at Test point 2 because it is positioned directly above the pipeline, corresponding to the S-wave low-energy zone directly above the source shown in Figure 3.

### 3.2. Localization of Buried PE Pipeline

**Figure 5:**
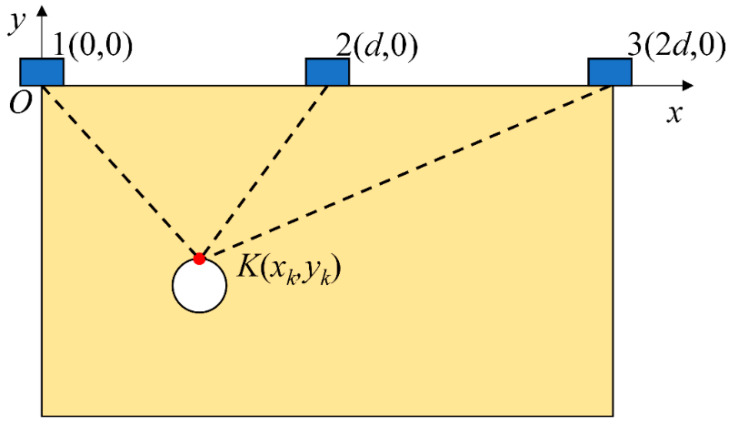
Schematic diagram of elastic wave migration stacking imaging.

This section employs the principle of elastic wave migration stacking imaging to perform localization simulation analysis for a buried PE pipeline. Using test point 1 in Figure 1 as the coordinate origin, we established the coordinate system shown in Figure 5. The coordinates of test point 2 and test point 3 are (d, 0) and (2d, 0), respectively. Point K(x_k_,y_k_) is one point in the soil domain. The propagation time t_ki_ (i = 1, 2, 3) for an elastic wave traveling from point K to each ground test point is:

(18) $$t_{ki}=\frac{\sqrt{{\left[x_{k}-(i-1)d\right]}^{2}+{y_{k}}^{2}}}{c}$$

where c is the propagation velocity of the elastic wave in the soil.

As established in Section 3.1, a low-energy zone for S-waves exists directly above the pipeline, which may cause deviations in imaging results. Qi Y et al. [31] demonstrated through simulation that the faster the wave speed, the higher the energy of the wave, and the easier it is to detect. Since P-wave velocity exceeds S-wave velocity, the P-wave signals identified in Figure 4 were selected for migration stacking imaging. Let *y_i_*(*t*) be the P-wave signal received at the *i*-th detection point, with arrival time *t_i_*. If *y_i_*(*t*) is shifted in the time domain by *t_i_*, the input signals at each detection point would coincide on the time axis, differing only in amplitude. Utilizing this assumption, the propagation time *t_k__i_* for a P-wave traveling from point *K*(*x_k_, y_k_*) to the *i*-th ground detection point needs to be calculated. The signal *y_i_*(*t*) at the *i*-th detection point is then shifted in the time domain by *t_k_*_i_, and the shifted signal becomes *y_k__i_*(*t*). Sum up all the shifted signals to obtain the stacked signal *F*(*t*), expressed as follows:

(19) $$F(t)=\sum_{i=1}^{n}y_{ki}(t)=\sum_{i=1}^{n}y_{i}\left(t_{i}+t_{ki}\right)$$

**Figure 6:**
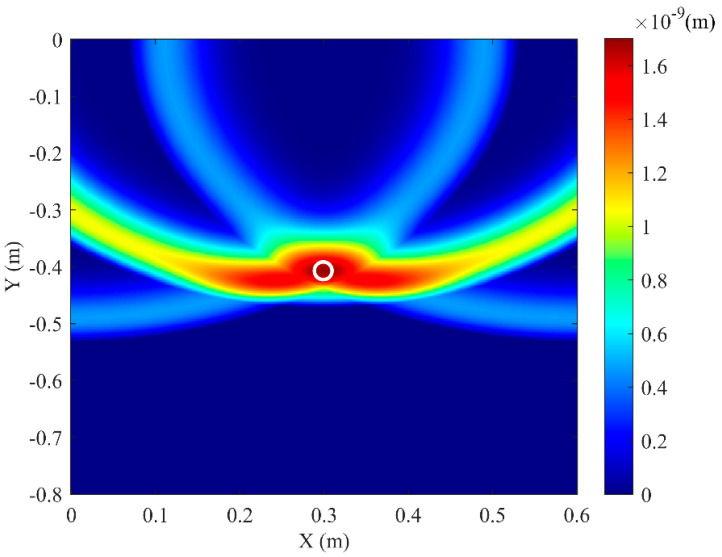
Simulation imaging result for pipeline actual vertex coordinates (0.3, −0.4).

**Figure 7:**
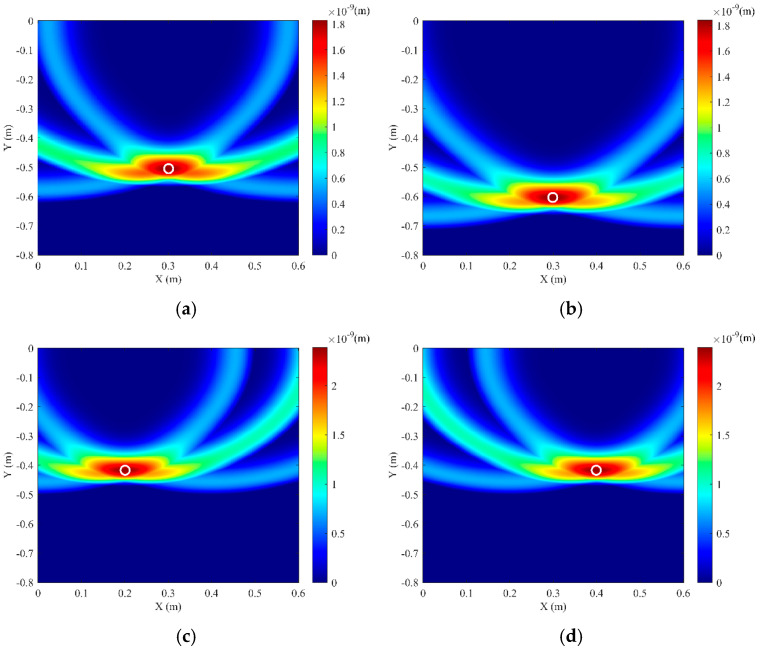
Simulation imaging results for different pipeline positions. (**a**) Pipeline actual coordinates (0.3, −0.5); (**b**) Pipeline actual coordinates (0.3, −0.6); (**c**) Pipeline actual coordinates (0.2, −0.4); (**d**) Pipeline actual coordinates (0.4, −0.4).

The soil domain shown in Figure 5 was divided into *n* square grids. The steps described above were repeated for all grid points within the soil domain, resulting in a two-dimensional amplitude matrix **M** with respect to the amplitude of *F*(t). The amplitude of signal *F*(*t*) reaches its maximum when point *K*(*x_k_, y_k_*) coincides with the top vertex of the pipeline. Using this method, two-dimensional stacking imaging localization was performed for a pipeline with the top vertex coordinates (0.3 m, −0.4 m), yielding the imaging result shown in Figure 6. It can be seen that the location with the peak amplitude value (darkest color) corresponds to the pipeline position. To better evaluate the localization accuracy for different burial positions, pipelines at four distinct locations were simulated. Appling the above imaging method, the position of these pipelines can be located, and the results are shown in Figure 7. To be more specific, the localization errors for the imaging results in Figure 6 and Figure 7 are listed in Table 1. Table 1 shows that the maximum horizontal position error is 0.667%, and the maximum depth error is 4.25%, indicating that this method can effectively locate the pipeline for various burial positions.

## 4. Experimental Verification

### 4.1. Experimental Setup

**Figure 8:**
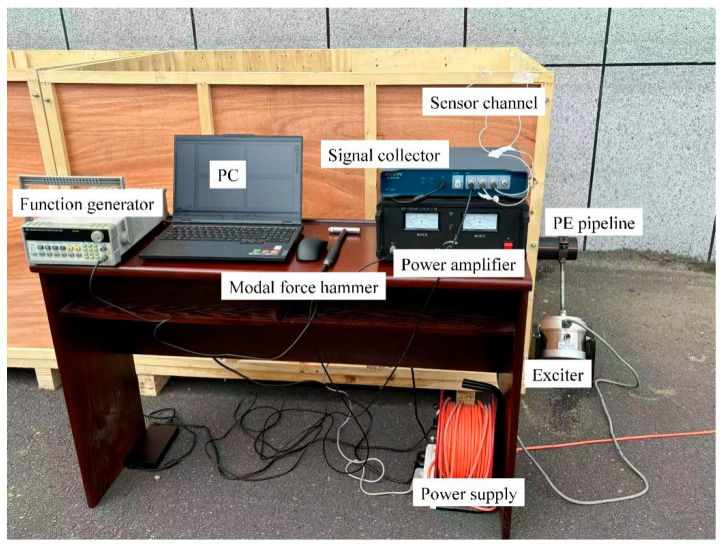
Schematic diagram of experimental setup.

**Figure 9:**
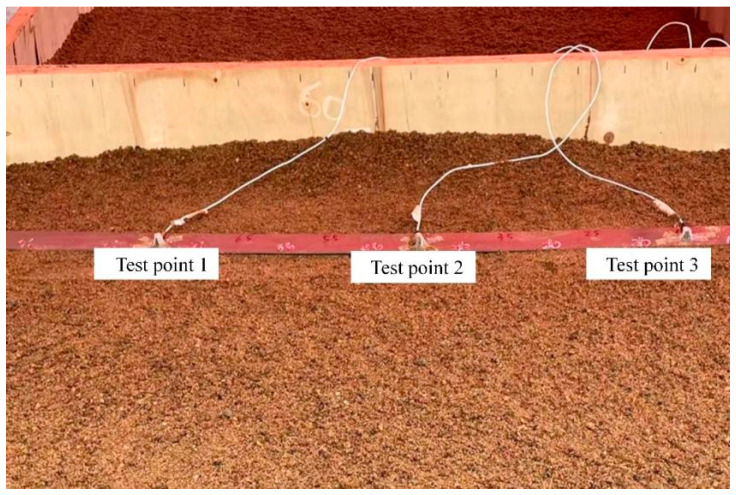
Schematic diagram of sensor arrangement.

To further validate the effectiveness of the elastic wave migration stacking imaging method for locating buried PE pipelines, a field localization experiment was conducted. The experimental setup, shown in Figure 8, includes a JZ-10 exciter, an SALC05KB modal impact hammer, an F05 digital synthesized function signal generator, a GF-100W power amplifier, an MI-7004 signal acquisition unit, three 353B15 acceleration sensors, a power supply unit, a PC, sand, and a rectangular wooden box containing a PE pipe. The sensor positions are shown in Figure 9, with the pipeline buried at a depth of 0.4 m. We did not use existing running PE pipelines, but chose to conduct experiments by independently burying PE pipelines. The reason for this is that the running PE pipeline is usually in a specific operating environment and conditions, which are not easy to change or adjust. This limitation hinders our adjustment of parameters such as pipeline burial depth, thereby affecting the reproducibility and accuracy of the experiment. In this study, we can flexibly control experimental conditions, such as soil materials and burial depth, to simulate pipelines under different conditions, ensuring the flexibility and accuracy of the experiments. The dimensions of the rectangular wooden box are 3 m in length, 1 m in height, and 1 m in length. The box is filled with sand, and the density test result of sand is 1985 kg/m^3^, which is same with the simulation.

### 4.2. Signal Processing and Wave Velocity Testing

**Figure 10:**
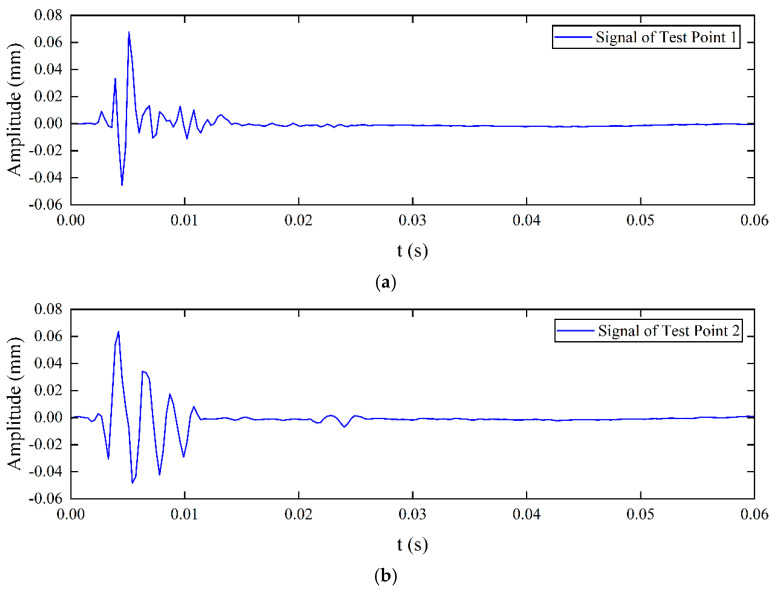

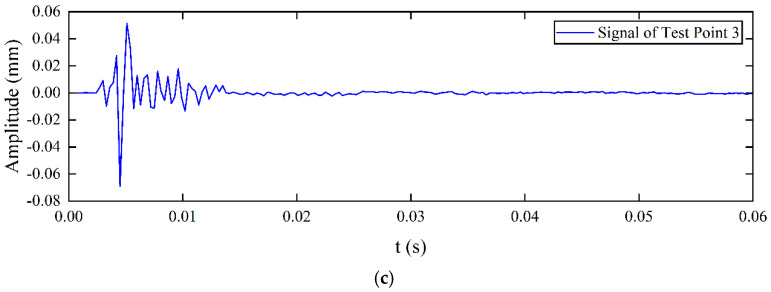
Signals measured at test points. (**a**) Signal of Test Point 1; (**b**) Signal of Test Point 2; (**c**) Signal of Test Point 3.

**Figure 11:**
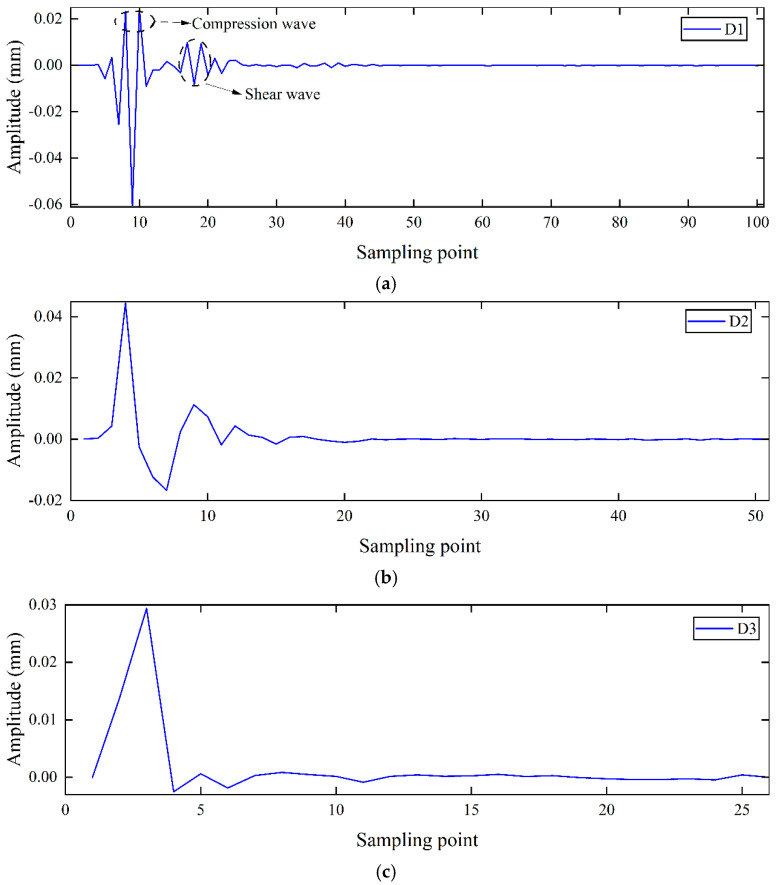

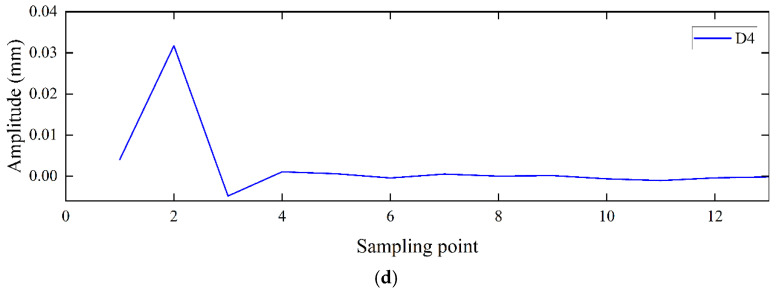
Wavelet decomposition (**a**) D1; (**b**) D2; (**c**) D3; (**d**) D4.

**Figure 12:**
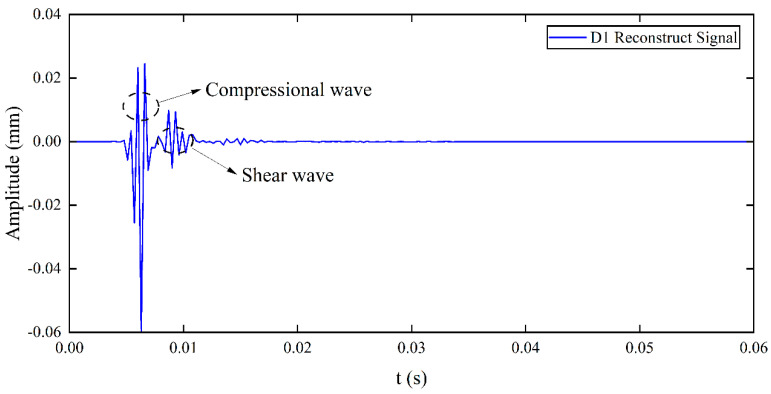
Signal reconstructed using D1 wavelet.

The signals measured at three test points during the experiment are shown in Figure 10. Due to soil inhomogeneity and external noise, the original signals were corrupted. Wavelet decomposition and reconstruction techniques were then employed for noise removal. Due to its compact support and orthogonality, Daubechies 7 (Db7, where 7 denotes the order) enables effective extraction of signal details during wavelet decomposition and achieves high fidelity in reconstruction, while providing a good balance between computational efficiency and noise resistance. Higher-order wavelets provide better reconstruction accuracy, reducing restoration errors and ensuring stable and accurate signal analysis. The wavelet decomposition of the signal in Figure 10a is shown in Figure 11. Since the D1 component in Figure 11a clearly represents the P-wave and S-wave signals, the signal is decomposed into four layers and reconstructed using the D1 component; the reconstructed signal is shown in Figure 12 [30].

**Figure 13:**
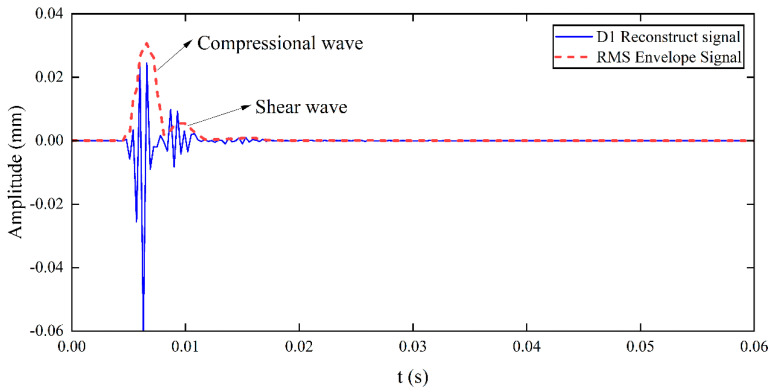
RMS upper envelope.

Although wavelet decomposition and reconstruction successfully suppressed noise and distinguished the P-wave from the S-wave in the experimental signals, the signals retained peak and valley features. Direct stacking of these signals could impair imaging accuracy. Therefore, to improve the accuracy of compressional wave migration imaging, peak envelope processing of the signal is necessary. The RMS envelope technique not only focuses on the maximum signal value but also averages instantaneous peaks, and considers the overall energy distribution of the signal, making it suitable for signals with large dynamic ranges. In this study, the RMS envelope technique is employed, with a window size set to 5, and the RMS upper envelope of the reconstructed signal is shown in Figure 13.

**Figure 14:**
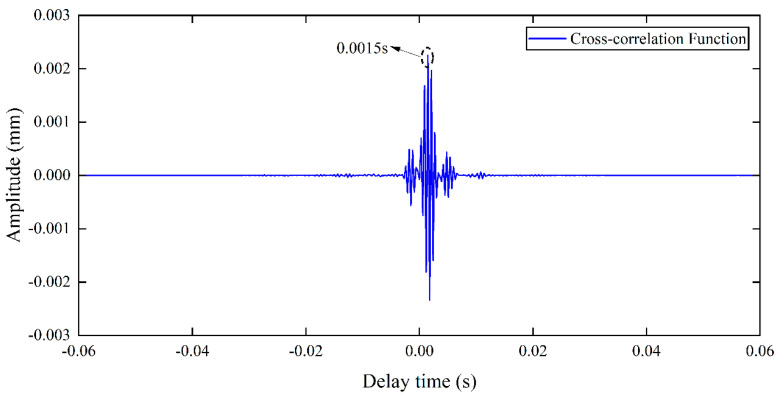
Cross-correlation function between Test Point 1 and Test Point 2.

To locate the position of PE pipeline, the P-wave velocity needs to be tested in the compressional wave migration imaging method. According to Equation (11), the reconstructed signals of Figure 10 (a) and (b) are, respectively, regarded as *y*_1_(*t*) and *y*_2_(*t*) in Equation (11), and the cross-correlation function is calculated as shown in Figure 14. It can be seen that the peak time of this cross-correlation function (time delay Δt) is 0.0015 s. In the experiment, the sensor spacing between test point 1 and test point 2 in Figure 9 is 0.3 m. Then the P-wave velocity can be calculated via Equation (13), yielding a result of 200 m/s. The P-wave velocity calculated using the cross-correlation principle is approximate, as factors such as sensor spacing accuracy and time delay resolution during the experiment can cause errors in the calculation of Δ*t*, thereby affecting the calculation of wave velocity.

### 4.3. Compressional Wave Imaging

**Figure 15:**
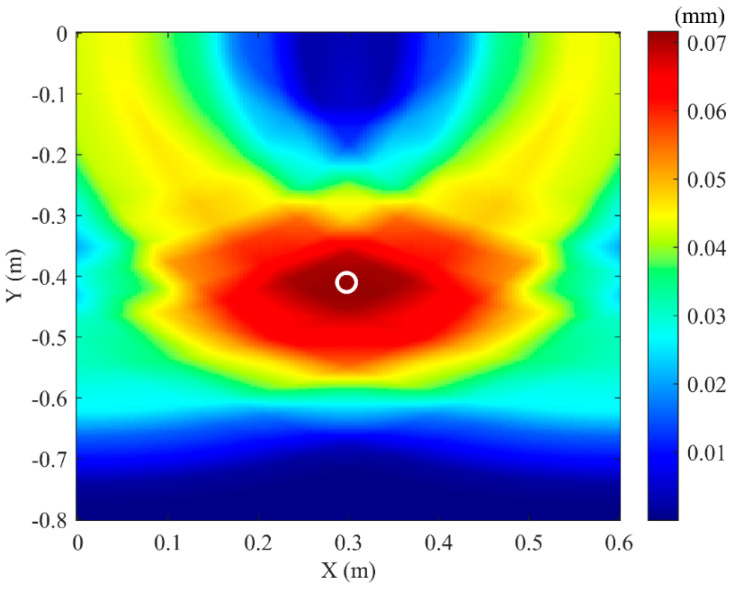
Experimental imaging result of buried pipeline.

**Figure 16:**
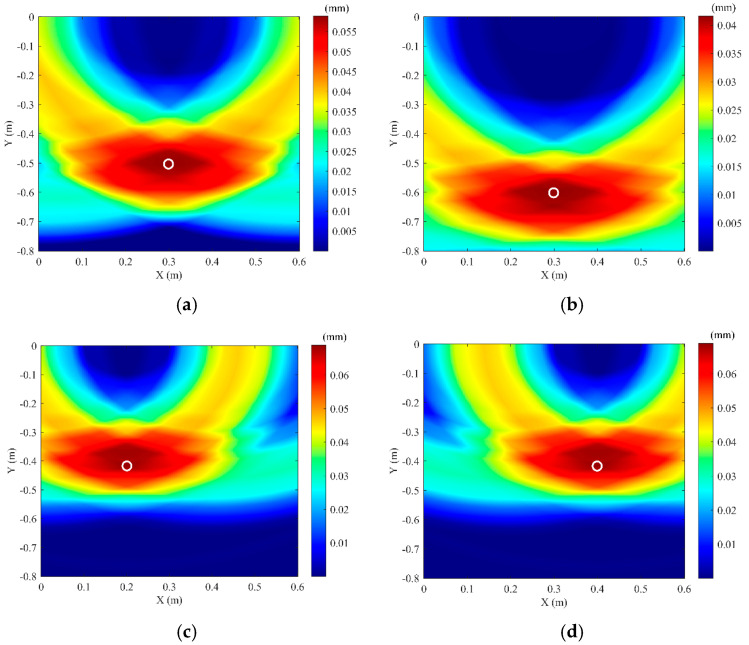
Experimental imaging results for different pipeline positions. (**a**) Pipeline coordinates (0.3, −0.5); (**b**) Pipeline coordinates (0.3, −0.6); (**c**) Pipeline coordinates (0.2, −0.4); (**d**) Pipeline coordinates (0.4, −0.4).

As introduced in Section 3.2, the compressional wave migration stacking imaging method is used. The S-wave in Figure 13 is removed, retaining only the P-wave. The signals from the three test points in Figure 10 undergo the same processing steps. Equations (18) and (19) are then applied to perform migration stacking imaging on the soil domain in the experiment. The imaging result is shown in Figure 15. The actual coordinates of the pipeline vertex are (0.3 m, −0.4 m), and the experimental imaging coordinates are (0.299 m, −0.409 m), resulting in a localization error of (0.33%, 2.25%). To better verify the proposed method, different pre-buried position of the pipeline are conducted in the experiments. The localization results are shown in Figure 16, and the error of experimental results are listed in Table 2. It can be seen that the horizontal localization error does not exceed 0.5%, and the depth localization error does not exceed 4.25%, indicating that this method can accurately locate the buried PE pipeline.

**Figure 17:**
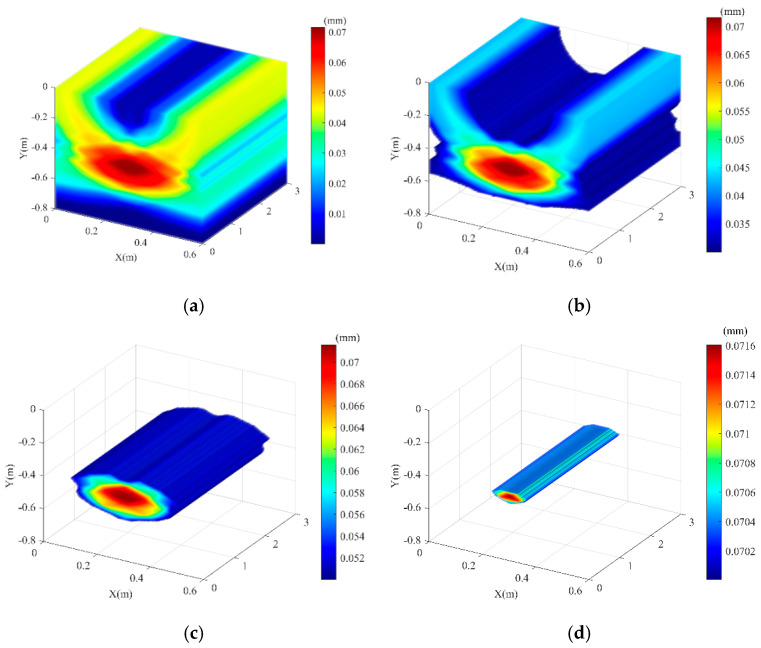
Three-dimensional reconstruction of pipeline direction for different thresholds. (**a**) Threshold 0; (**b**) Threshold 0.03; (**c**) Threshold 0.05; (**d**) Threshold 0.07.

To further visualize the direction of the buried pipeline underground, three-dimensional imaging was reconstructed by axially stretching multiple two-dimensional cross-sections, as shown in Figure 17. By setting different thresholds for the stacking coefficient, the imaging results display only regions exceeding the threshold. Figure 17 shows the results for stacking coefficient thresholds of 0, 0.03, 0.05, and 0.07. Clearly, directional information of the underground pipeline can be obtained from the three-dimensional reconstructed images when the stacking coefficient threshold is selected reasonable. Experimental results demonstrated that the proposed stacking imaging method can effectively detect the horizontal coordinates, vertical depth, and direction of underground pipelines. Furthermore, Cui X et al. [32] effectively determined the direction of PE gas pipelines in service through 3D reconstruction, and distinguished them from other impurities in the soil such as rocks, bricks, trees, etc. This provides ideas for subsequent research in this paper.

## 5. Conclusions

To address the issues of signal interference and insufficient accuracy in buried PE pipeline localization techniques, this paper proposed a localization method based on compressional wave migration stacking imaging. The theoretical analysis of the proposed method is introduced, and the simulation model of a buried pipeline is established. The simulation results of this localization method demonstrate an excellent accuracy. To verify the localization method, field experimental tests are conducted, and the experimental results have a good agreement with the simulation results. Some conclusions are drawn as follows:

1. The wave velocity equations for P-wave and S-wave are derived, and P-wave velocity is significantly higher than S-wave velocity, which is verified through simulations. The compressional wave migration stacking imaging algorithm can clearly extracted pipeline position information, and the simulation localization results show horizontal localization errors below 0.667% and depth errors below 4.25%, demonstrating the feasibility of the proposed method.

2. The pipeline excitation technology is employed to enhance the acoustic signal strength while avoiding interference from surface waves and direct waves. Wavelet decomposition, reconstruction, and RMS envelope techniques are utilized to remove noise interference and improve imaging accuracy. The P-wave velocity in the experimental soil can be estimated using cross-correlation theory, providing a reliable basis for compressional wave migration imaging.

3. Field experimental results showed that the compressional wave migration stacking imaging method achieved horizontal localization errors below 0.5% and depth errors not exceeding 4.25%. The three-dimensional reconstruction technology can intuitively display the direction of the pipeline, verifying the reliability of the proposed method. However, since the experiment was conducted in a rectangular wooden box, it can only indicate that the method is effective in the experimental environment of this paper. This method can provide a reference for pipeline positioning in actual field experiments, but the effectiveness of positioning still needs further research.

Although the method proposed in this paper can accurately locate buried PE pipelines, there may still be gaps in practical applications. For buried pipelines in the actual environment, the propagation of elastic waves is constrained not only by geometric factors but also by the material properties of the medium and environmental conditions, especially under complex geological conditions. The current research is still in its initial stage, primarily focusing on the compressional wave migration imaging algorithm. Future research will further optimize the computational efficiency of the algorithm. Meanwhile, considering actual geological conditions, the influence of multi-physics coupling (such as temperature and humidity) on elastic wave propagation will be explored to improve localization accuracy and stability in extreme environments.

## Figures and Tables

**Table 1:** Simulation localization errors of elastic wave migration stacking imaging.

No.	Pipeline Actual Coordinates	Simulated Localization Coordinates	Error
1	(0.3, −0.4)	(0.299, −0.407)	(0.333%, 1.75%)
2	(0.3, −0.5)	(0.301, −0.505)	(0.333%, 1%)
3	(0.3, −0.6)	(0.298, −0.604)	(0.667%, 0.667%)
4	(0.2,−0.4)	(0.2007,−0.417)	(0.35%, 4.25%,)
5	(0.4,−0.4)	(0.3993,−0.417)	(0.175%, 4.25%)

**Table 2:** Experimental localization errors of elastic wave migration stacking imaging.

No.	Actual Pre-Buried Pipeline Coordinates	Experimental Imaging Coordinates	Experimental Error
1	(0.3, −0.5)	(0.299, −0.503)	(0.333%, 0.6%)
2	(0.3, −0.6)	(0.299,−0.6015)	(0.333%,0.25%,)
3	(0.2,−0.4)	(0.201,−0.417)	(0.5%,4.25%)
4	(0.4,−0.4)	(0.399,−0.417)	(0.25%,4.25%)

## Data Availability

The raw data supporting the conclusions of this article will be made available by the authors on request.
